# Papillary Ependymoma WHO Grade II of the Aqueduct Treated by Endoscopic Tumor Resection

**DOI:** 10.1155/2009/434905

**Published:** 2009-08-24

**Authors:** Andreas M. Stark, Heinz-Herrmann Hugo, Arya Nabavi, H. Maximilian Mehdorn

**Affiliations:** Klinik für Neurochirurgie, Im Universitätsklinikum Schleswig-Holstein, Campus Kiel, 24105 Kiel, Germany

## Abstract

Papillary ependymoma is a rare tumor that may be located along the ventricular walls or within the spinal cord. We report the case of a 54-year-old patient with a papillary ependymoma WHO grade II arising at the entrance of the aqueduct. The tumor caused hydrocephalus. The tumor was completely removed via a right-sided endoscopic approach with restoration of the aqueduct. The free cerebrospinal fluid passage through the aqueduct was not only visualized by endoscopy but also controlled by intraoperative high-field magnetic resonance imaging. Therefore, an additional endoscopic third ventriculostomy was unneccessary.

## 1. Introduction

 Tumor related obstructive hydrocephalus is a frequent event. In most cases however, the cerebro spinal fluid (CSF) pathway is compressed by an intraparenchymal lesion itself or by the surrounding edema (glioma, brain metastases). In the minority of cases, tumors arise within the ventricular walls (choroid plexus papilloma, intraventricular meningioma, ependymoma) [1]. In this condition, endoscopic and microsurgical techniques can be used either alone or in combination to remove the tumor and restore the cerebro-spinal fluid (CSF) pathway [2–4]. Surgical intervention might be followed by chemo- and/or radiotherapy. A specific subset of hydrocephalus-inducing tumors is tectal gliomas which arise around the aqueduct. This tumor type usually requires only restoration of CSF flow (achieved by endoscopic third ventriculostomy). Radiotherapy can be applied in cases of tumor progression [5]. We report a case of hydrocephalus caused by a small lesion occluding the entrance of the aqueduct which turned out to be a papillary ependymoma WHO grade II. The tumor was removed via an endoscopic approach with restoration of the aqueduct. The operative result was controlled via intra-operative magnetic resonance imaging (MRI).

## 2. Case Presentation

### 2.1. Clinical Presentation

**Figure 1:**
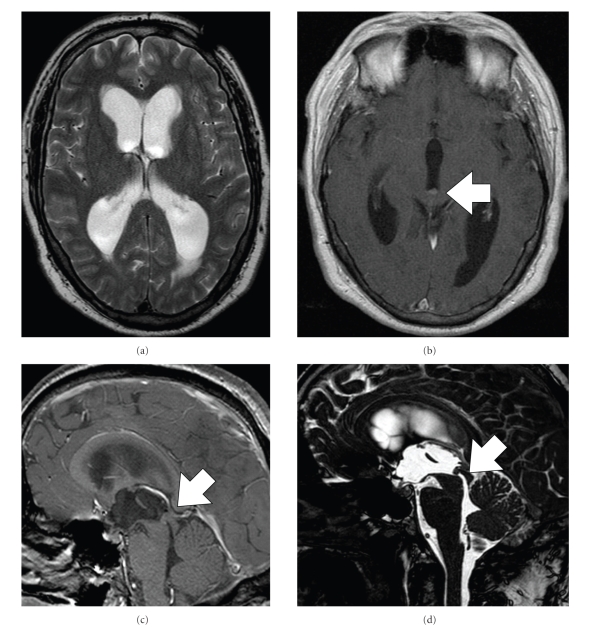
(a) T2-weighted MRI showing marked hydrocephalus, T1-weighted contrast enhanced MRI reveals a small lesion tumor at the cranial end of the aqueduct in axial (b) and sagittal view (c) and TRUFI sequences (sagittal thin T2-weighted images, (d)).

**Figure 2:**
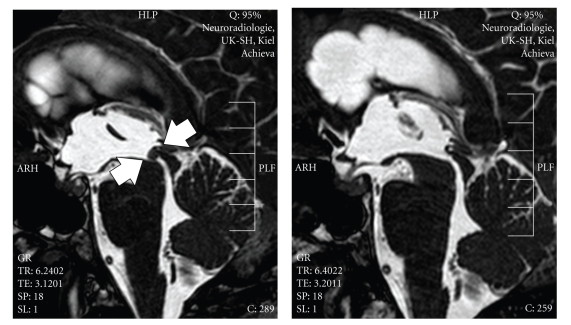
T2-weighted sagittal MRI (left) before and (right) after endoscopic tumor removal with restoration of the aqueduct. The tumor is marked by arrows.

A 54-year-old man presented to our clinic with progressive headache, visual disturbance, and, gait impairment since 9 months. Neurological examination revealed marked ataxia, no further neurological deficits. Magnetic resonance imaging (MRI) of the brain revealed obstructive hydrocephalus (Figure 1) caused by a small tumor at the cranial entry of the aqueduct (Figure 2(a)). 

### 2.2. Treatment

**Figure 3:**
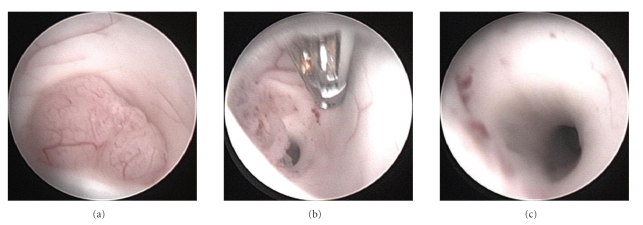
Intraoperative endoscopic images of the tumor at the entrance of the aqueduct before (a), within (b), and after tumor resection (c).

 The patient underwent tumor resection via a rigid neuroendoscope. Neuronavigation was used as an aid for exact burrhole placement. We sought to access the aqueduct as well as, possibly, the floor of the third ventricle for third ventriculostomy. The burrhole was placed right frontal approximately 5 cm in front of the coronal suture. Intraoperatively, the tumor was found to be smooth and moderately vascularized (Figure 3(a)). The lesion was coagulated with bipolar coagulation and than removed using forceps (Figures 3(b) and 3(c)). Intraoperative high-field MRI revealed a free CSF passage of the aqueduct (Figure 2(b)). As a consequence of this finding, endoscopic third ventriculostomy was not performed. A ventricular drainage was inserted through the burr hole without connection to a drainage system.

### 2.3. Histopathology

**Figure 4:**
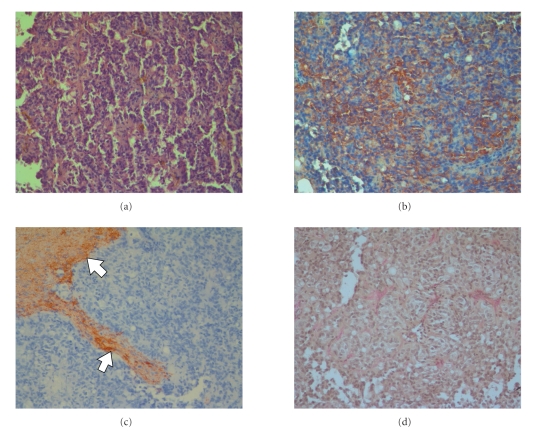
Papillary ependymoma: HE (a) S-100 protein (b) GFAP (c), Elastica-van-Giessen staining (d).

Histopathological examination showed a papillary tumor with increased cellularity. The chromatin of the round nuclei was fine granular and the cytoplasm was eosinopilic. There were small perivascular anuclear zones with fibrillary processes. Mitoses were absent (Figure 4(a)). Immunohistochemically the tumor cells were positive for S-100 Protein (Figure 4(b)) but negative for GFAP (Figure 4(c)). Elastica-van-Giesson staining showed reticulin fibers (Figure 4(d)). The Ki67 labelling index (MIB 1-antibody) approached 5%. The tumor was diagnosed as papillary ependymoma WHO grade II. 

### 2.4. Postoperative Course

Postoperatively, the patient developed transient headache which was treated by CSF diversion via the ventricular drainage and an additional lumbar drainage. Hypothetically, we sought to relief transient hydrocephalus due to poor resorption in long-standing hydrocephalus. Herein, the symptoms resolved over few days and the drains could be removed. He showed also diplopia which was intermittend at first but then remained stable over the follow-up period of 15 months. Postoperative MRI revealed no spinal drop metastases. 

## 3. Discussion

 Papillary ependymoma is a rare variant of ependymoma. The differential diagnosis includes choroid plexus papilloma, papillary meningioma and metastatic papillary carcinoma [1]. Ependymomas usually correspond to WHO grade II with a ten-year survival rate of approximately 45% in adults. Anaplastic ependymoma (WHO grade III) is associated with unfavorable clinical outcome [1]. Other ependymoma variants are cellular, clear cell and tanycytic types. Furthermore, ependymomas with lipomatous differentiation, giant cell formation, extensive vacuolation, melanotic differentiation, signet ring cell appearance and ovarian localization have been reported. Supratentorial location has a better outcome than infratentorial location. Spinal location of ependymoma is also associated with a better prognosis [1, 6]. 

 Reviewing the literature, we found 4 articles fulfilling our request (PubMed, “papillary ependymoma” in the title, human) which were published between 1963 and 1996. In 1996, Park et al. reported two cases of large papillary ependymoma in the left lateral ventricle and the pineal region, both cases were associated with hydrocephalus [6]. In one case reported in Italian in 1965, “encephalitic symptomatology due to a papillary ependymoma of the 3rd ventricle” was reported [7]. An abstract is not available. Other locations reported are the cervical spinal cord [8], and the cauda equine [9]. In the latter case, metastasis to other organs has been reported. 

 In our case, a small papillary ependymoma was located at the caudal entry of the aqueduct and caused hydrocephalus. The patient was treated by endoscopic tumor removal with restoration of the aqueduct. Intraoperative MRI was used to document macroscopically total tumor resection and restoration of the aqueduct. In such cases with small tumors arising along the ventricular walls, macroscopically total tumor removal should be achieved. In case of failed or questionable restoration of the aqueduct, endoscopic third ventriculostomy (ETV) should be performed. Herein, intraoperative MRI can be helpful in the decision whether ETV should be performed or not. Notable, patients should be followed postoperatively for possible secondary occlusion of the aqueduct due to adhesions. In this case, ETV might be necessary afterwards. Insertion of a ventricular-peritoneal shunt is no longer an alternative as first treatment in these patients [2, 3]. We chose a right frontal burrhole approximately 5 cm before the coronal suture. Herein we sought to access the aqueduct and, if needed, the floor of the third ventricle. 

 In conclusion, papillary ependymoma is a rare tumor that arises along the CSF-pathway and may cause hydrocephalus. MRI is the key diagnostic tool to define the cause of hydrocephalus. Surgery, either open microsurgical resection or endoscopic resection can erase the tumor. In small tumors, endoscopy is the treatment of choice. If the aqueduct can not be restored or restoration is questionable, endoscopic third ventriculostomy should be performed. Neuronavigation can be helpful in these case to estimate the exact angle of endoscopy entrance. Histological examination of the surgical specimens is the key to diagnosis. Even if the tumor correnponds to WHO grade II, metastasis to other organs has been reported.
